# Response of the microbial community to phosphate-solubilizing bacterial inoculants on *Ulmus chenmoui* Cheng in Eastern China

**DOI:** 10.1371/journal.pone.0247309

**Published:** 2021-02-25

**Authors:** Juan Song, LiJing Min, JunRong Wu, Qingfang He, FengMao Chen, Yang Wang

**Affiliations:** 1 Collaborative Innovation Center of Sustainable Forestry in Southern China, College of Forestry, Nanjing Forestry University, Nanjing, China; 2 Institute of Forest Protection, College of Forestry, Nanjing Forestry University, Nanjing, China; 3 College of Life Science, Huzhou University, Key Laboratory of Vector Biology and Pathogen Control of Zhejiang Province, Huzhou, Zhejiang, China; 4 Department of Biology, University of Arkansas at Little Rock, Little Rock, Arkansas, United States of America; COMSATS University Islamabad - Abbottabad Campus, PAKISTAN

## Abstract

Phosphate-solubilizing bacteria (PSB) have beneficial effects on plant health and soil composition. To date, studies of PSB in soil have largely been performed under field or greenhouse conditions. However, less is known about the impact of introducing indigenous PSB in the field, including their effects on the local microbial community. In this study, we conducted greenhouse and field experiments to explore the effects of the addition of indigenous PSB on the growth of Chenmou elm (*Ulmus chenmoui*) and on the diversity and composition of the bacterial community in the soil. We obtained four bacterial isolates with the highest phosphate-solubilizing activity: UC_1 (*Pseudomonas* sp.), UC_M (*Klebsiella* sp.), UC_J (*Burkholderia* sp.), and UC_3 (*Chryseobacterium* sp.). Sequencing on the Illumina MiSeq platform showed that the inoculated PSB did not become the dominant strains in the *U*. *chenmoui* rhizosphere. However, the soil bacterial community structure was altered by the addition of these PSB. The relative abundance of *Chloroflexi* decreased significantly in response to PSB application in all treatment groups, whereas the populations of several bacteria, including *Proteobacteria* and *Bacteroidetes*, increased. Network analysis indicated that *Chloroflexi* was the most strongly negatively correlated with *Proteobacteria*, whereas *Proteobacteria* was strongly positively correlated with *Bacteroidetes*. Our findings indicate that inoculation with PSB (UC_1, UC_M, UC_J, and UC_3) can improve the growth of *U*. *chenmoui* and regulate its rhizosphere microbial community. Therefore, inoculation with these bacterial strains could promote the efficient cultivation and production of high-quality plant materials.

## Introduction

*Ulmus chenmoui* Cheng (Ulmaceae) is an endangered tree species that is endemic to eastern China, where it is mainly found on Langya Mountain in Anhui Province and Huashan Mountain in Jiangsu Province [1, 2]. Adult *U*. *chenmoui* tree populations have seriously declined due to their economic value [3], and young tree populations have also dramatically decreased due to the destruction of their natural habitat. Long-term artificial changes to this habitat can alter the growth and severely damage the soil microbial community structure of *U*. *chenmoui*. Therefore, the protection and utilization of endangered and endemic species requires an effective strategy possibly involving the use of functional bacteria [4].

Phosphate-solubilizing bacteria (PSB) play a critical role in the biogeochemical cycling of soluble and insoluble P in ecosystems, as they convert insoluble P into soluble P, which is available to plants [5–7]. Some PSB even show potential as biocontrol agents against some plant pathogens [7]. To date, PSB have been isolated from various environments, including plant rhizospheres, solid waste compost, and metal-contaminated soils [8–10]. Although PSB have been widely studied and applied, few studies have characterized PSB from the rhizosphere of *U*. *chenmoui* Cheng.

Plant and soil bacterial communities are intimately related [11–14]. Rhizosphere microbes play fundamental roles in plant growth and health [15–17], while plants help sequester atmospheric carbon, produce oxygen, and stabilize the soil to prevent excessive erosion [18–21]. Furthermore, a growing body of studies have shown that plant roots shape the composition of the root microbiota [22–24]. Plant–microbe interactions thus have important implications for ecosystem function at broad scales, including productivity and terrestrial communities [25, 26]. However, few studies have associated changes in the rhizosphere microbiome of *U*. *chenmoui* with the introduction of PSB.

The application of biofertilizers represents a sustainable pathway to improve plant growth [27, 28], whereas current agricultural practices have negatively affected soil microbial composition and biodiversity [29] and are not sustainable. The inoculation of rhizobia in soil increases the phylotype richness of bacterial communities and affects the functional capabilities of soil microbial communities [30]. The inoculation of beneficial bacteria into soil planted with tomato (*Solanum lycopersicum*) altered the composition of the microbial community in the rhizosphere [31]. Therefore, additional attention should be given to the effects of exogenous microbial inoculants, including PSB applied to calcareous soils, on the diversity and composition of soil bacterial communities. The PSB from *U*. *chenmoui* may provide an economical, eco-friendly way to improve the availability of phosphorus in the soil for use by this tree species.

In the current study, we (1) investigated the native PSB from the *U*. *chenmoui* Cheng rhizosphere and their effects on the growth of *U*. *chenmoui* seedlings; (2) determined whether and how the application of PSB influences the diversity and composition of the indigenous bacterial community in soil in the field; and (3) identified microbial groups that could potentially help provide specific environmental conditions suitable for the endangered tree species *U*. *chenmoui*. The results of this study should facilitate the use of these strains in sustainable forestry and the optimization of the composition of the rhizosphere bacterial community.

## Materials and methods

### Soil sampling to identify PSB in the *Ulmus chenmoui* rhizosphere

Soil samples were collected from the rhizosphere of *U*. *chenmoui* Cheng. Eight samples were collected from two different environments, Mount Langya in Anhui province (118°29.3741’ E, 32°28.6448’ N) and the Nanjing Zhongshan Botanical Garden in Nanjing province (118°83.6244’ E, 32°06.0579’ N), China. This research does not involve animal subjects or any other endangered species. All locations are public lands that do not require specific permission for public funded research in China. We selected our target tree species on the open public land and took 1kg of its rhizosphere soil sample. In addition, there are no endangered species near the selected tree. Our target tree species are older than 30 years, the collection of rhizosphere soil samples did not have any impact on the health of the tree species. The three sampling sites from Mount Langya were ≥ 30-year-old elm forests (with healthy trees), and the three sampling sites from Nanjing Zhongshan Botanical Garden were ≥ 35-year-old elm forests (with healthy trees). The sampling did not follow a regular grid because we wanted to characterize the PSB that were closely associated with this dominant tree species. Each soil sample was pooled in a sterile plastic bag and transported to the laboratory on ice. Upon arrival in the laboratory, the samples were sieved (2 mm) and stored at 4°C and -20°C until analysis.

### Isolation of PSB and assessment of phosphate solubilization activity

Bacteria were isolated from the rhizosphere soil of *U*. *chenmoui* Cheng from the two sampling sites. Briefly, 1 g of soil sample was dissolved in 9 mL of sterile water (pH 7.0) and subjected to sequential 10-fold dilutions. Aliquots (100 μL) of the sixth dilution (10^−6^) were spread on nutrient agar plates and incubated for 1 day at 28°C. Bacterial colonies were subsequently isolated and subcultured to obtain individual strains. All bacterial isolates were screened for phosphate solubilization activity on National Botanical Research Institute’s Phosphate (NBRIP) agar medium [32] or in egg yolk agar [33]. Relative phospholipase activity was calculated as (diameter of halo/diameter of colony)×100. Gram staining of bacteria was performed as previously described [34]. Bacterial isolates were then grouped based on phenotypic features (i.e., color, shape, motility, growth rate, and colony morphology) and stored in a refrigerator at 4°C until analysis. For long-term maintenance, a subset of the isolates was stored in nutrient broth (NB) containing 20% glycerol at −80°C.

Quantitative estimations and comparisons of the phosphate solubilization ability of the bacterial isolates were conducted using cultures grown in NBRIP or Pikovskaya (PVK) broth medium, and all tests were conducted in triplicate. To determine the ability of inorganic P to be solubilized in NBRIP broth medium, strains with an inoculum size of 1.5% (OD_600_ = 0.5, v:v) were inoculated into a 100-mL Erlenmeyer flask containing 50 mL of NBRIP medium and incubated on a rotary shaker at 180 rpm and 30°C for 4 days. The broth was centrifuged at 1,003 g for 10 min and its P concentration measured [35] using the phosphomolybdate method [36, 37]. A separate broth medium inoculated with sterile Milli-Q water served as the control.

Similarly, we conducted quantitative estimation and comparison of the phosphate solubilization ability of organophosphorus-degrading bacteria isolates in PVK broth medium. A 2-mL aliquot of an overnight culture of each organophosphorus-degrading bacterial isolates was inoculated in 100 mL of PVK broth in three replicates. All cultures were incubated at 30 ± 2°C. The amount of P released in the broth was estimated at 4 days of culture from triplicate flasks compared with a set of un-inoculated controls. The amount of soluble P was determined by the phosphomolybdic blue color method [38].

Isolates UC_1, UC_M, UC_J, and UC_3 had the highest phosphate solubilization activity and were therefore selected for further study. All tests were conducted in triplicate.

### Genomic DNA extraction from bacterial isolates and PCR amplification

Genomic DNA was extracted from the four highly effective PSB isolates using the phenol/chloroform method [39], and the 16S rRNA gene was amplified from each bacterial isolate by polymerase chain reaction (PCR) using universal bacterial primers 27F (5’-AGAGTTTGATC(C/A)TGGCTCAG-3’) and 1492R (5’-GGTT ACCTTGTTACGACTT-3’) [40]. The PCR mixture contained 2× Taq PCR Master Mix (Genomics BioScience and Technology) and 4 μL of primers concentration. 16S rDNA was amplified by subjecting the samples to initial denaturation at 94°C for 3 min, followed by 35 cycles of 94°C for 30 s, 52°C for 30 s, and 72°C for 1.5 min, and a final extension at 72°C for 7 min, after which the samples were held at 4°C. The PCR products were confirmed by gel electrophoresis on a 1% agarose gel and purified using a GeneJET Gel kit (Thermo Scientific, EU).

The 16S rRNA sequences were analyzed by performing a similarity search of the GenBank database at the National Center for Biotechnology Information using the BLAST-N program (http://www.ncbi.nlm.nih.gov). The gene sequences were aligned, and a phylogenetic tree was constructed by the neighbor-joining method with 1,000 bootstraps using MEGA 7.0 software [41].

### HPLC analysis of organic acid contents

The cultures of bacteria incubated with NBRIP broth as described above were centrifuged at 10,000 rpm for 10 min to pellet the bacterial cells. The supernatant was passed through a 0.22-μm filter membrane, and 20-μL aliquots of filtrate were injected into an HPLC system (PerkinElmer Series 200, USA) equipped with a UV/Vis detector. For analysis, 0.05 mol·L^−1^ KH_2_PO_3_ (pH 3.2) was applied as the mobile phase at a constant (isocratic) flow rate of 1.0 mL/min and a column temperature of 30°C. The retention time of each signal was recorded at a wavelength of 214 nm. Finally, HPLC profiles of the culture filtrates were analyzed via comparison with the elution profiles of standard organic acids [42].

### Inoculation of *U*. *chenmoui* plants with PSB

#### *U*. *chenmoui* pot experiment

*U*. *chenmoui* seeds were surface sterilized successively with 75% ethanol for 5 min and 1% sodium hypochlorite for 5 min and rinsed three times with sterile water. The seeds were sown in plastic pots (8-cm diameter and 10-cm height) containing 400 g of sterilized soil. The four PSB isolates (UC_1, UC_M, UC_J, and UC_3) were incubated (2.0 × 10^8^ CFU/mL) in pots. Each experiment was replicated 10 times per treatment. *U*. *chenmoui* plants were grown under natural lighting and a day/night temperature of 24/20 °C. After 1, 45, and 90 days, plants were sampled and the plant height and ground diameter were determined.

#### *U*. *chenmoui* field experiment

The experiment was conducted at the Mount Langya Experimental Farm (32°15′54″ N, 118°18′35″ E). The site has a subtropical monsoon climate with a mean annual temperature of 15.2°C and average annual precipitation of 1050 mm. Two-month-old *U*. *chenmoui* plants were used in the field experiment. 1,500 mL of 2.8 × 10^8^ CFU/mL of the UC_1, UC_M, UC_J, or UC_3 isolates was added to the *U*. *chenmoui* seedlings. Other seedlings were used for the untreated control (CK). The experimental fields were divided into 100 identical plots (1 × 1 m). A randomized block design was used with 20 replicates. The experimental farmland was managed using standard procedures during the *U*. *chenmoui* growing season. Trees were randomly sampled, and plant height and ground diameter were measured at 60 and 120 days after inoculation.

### Colonization of Rif-resistant mutants with PSB in the rhizosphere of *U*. *chenmoui* Cheng

The colonization of PSB was determined using the antibiotic labeling method with minor modifications [43] Rifampicin (Rif)-resistant mutants of the four PSB strains were generated by culturing the isolates on medium containing Rif (350 μg/mL) in the dark for 2 days at 28°C. Stable mutant PSB strains were selected, and the ability of the four mutant PSB strains (UC-M, UC-3, UC-J, and UC-1) to colonize *U*. *chenmoui* roots was assessed. The Rif-resistant mutant PSB strains were inoculated (1.0 x 10^8^ CFU/mL or 2.8 × 10^8^ CFU/mL) onto *U*. *chenmoui* roots in the natural environment in August. At 1, 15, and 30 days after inoculation, the Rif mutant cells were cultured on a plate containing rifampicin (350 g/mL). Rifampicin-resistant isolates with phenotypic features (i.e., color, shape, motility, and colony morphology) consistent with previously induced rifampicin (Rif)-resistant mutants were counted.

### DNA isolation and Illumina MiSeq of microbial communities in the field

To isolate soil bacterial DNA, 0.1 g of soil from each treatment in the field experiment was used. We normalized the isolated samples to a concentration of 10 ng/μL. Genomic DNA was extracted from the samples using a FastDNA Spin Kit for Soil (MP Biomedicals, LLC, Solana Beach, CA, catalog number 116560200), and primers specific for the V4 region of 16S rRNA (338F: 5′-ACTCCTACGGGA GGCAGCAG-3′; and 806R: 5′-GGACl ACHVGGGTWTCTAALT-3′) were used for PCR. The samples were barcoded separately to enable multiplex sequencing. The PCR amplifications were conducted in 20 μL master mix consisting of 4 μL 5× FastPfu Buffer, 2 μL dNTPs (2.5 mM), 0.8 μL forward primer (5 μM), 0.8 μL reverse primer (5 μM), 0.4 μL FastPfu Polymerase, 0.2 μL BSA, and 10 ng template DNA. The amplification process was conducted according to the following procedure: 3 min of initial denaturation at 95°C followed by 25 cycles of denaturing at 95°C, annealing at 59°C for 30 s, and extension at 72°C for 45 s, and a final extension at 72°C for 10 min. Three PCR products per sample were pooled to mitigate reaction-level PCR biases. The PCR products were purified using a QIAquick Gel Extraction Kit (Qiagen, Germany), quantified via quantitative PCR, and sequenced at Allwegene Company, Beijing. Deep sequencing was performed on the MiSeq platform at Allwegene Company (Beijing). After the run, image analysis, base calling, and error estimation were performed using Illumina Analysis Pipeline Version 2.6.

Data from the sequencing run have been deposited in the Sequence Read Archive at NCBI under accession number PRJNA681431.

### Processing of sequencing data and statistical analysis

Raw fastq files were demultiplexed, quality-filtered with Trimmomatic [44], and merged using FLASH [45] with the following criteria: (i) 300-bp reads were truncated at any site with an average quality score <20 over a 50-bp sliding window; (ii) sequences with overlaps > 10 bp were assembled; (iii) sequences of each sample were separated according to barcodes (exact match) and primers (exact match), and reads containing ambiguous characters were removed; (iv) chimera checking was conducted using customized USEARCH [46] software. Reads that could not be assembled were discarded, and high-quality clean tags were obtained. Sequences were de-replicated, discarding singletons, and clustered into operational taxonomic units (OTUs) at a similarity level of 97% [47] to generate rarefaction curves [48] and to calculate the richness and diversity indices [49]. Finally, the taxonomy of each 16S rRNA gene sequence was analyzed using the Ribosomal Database Project (RDP) Classifier tool [50] against the Silva (SSU123) 16S rRNA database with a confidence threshold of 70%.

To analyze microbial α-diversity, observed richness (number of OTUs) and Shannon diversity index were estimated based on OTU abundance matrices rarefied to the lowest number of sequences. To compare the membership and structure of the communities in different samples, heat maps were generated from the top 20 OTUs using Mothur. The alpha diversity metrics were calculated using Mothur (version v.1.30.1, *collect*.*single command*), while beta diversity measures and other analyses were calculated using the QIIME [51] and R packages. To examine the similarity between different samples, clustering analysis and principal component analysis (PCA) were performed based on the OTU information from each sample using an R package.

Co-occurrence analysis of *Ulmus chenmoui* bacterial communities was performed using Sparse Correlations for Compositional (SparCC) data with 500 bootstraps to estimate the *p* value. In total, 98% of rarefied OTUs were collapsed up to the phyla level. Non-significant correlations (*p* < 0.05) were excluded.

All experimental treatments were conducted in triplicate, and the results were expressed as the means ± standard deviation. Data were statistically analyzed using SPSS (Statistical Package for the Sciences System) version 17, and the results are shown as the mean values. Differences between mean values were identified by one-way analysis of variance (ANOVA). *P* ≤ 0.05 was considered statistically significant.

## Results

### Isolation of rhizosphere bacteria and the phosphate-solubilizing ability of four selected strains

**Fig 1:**
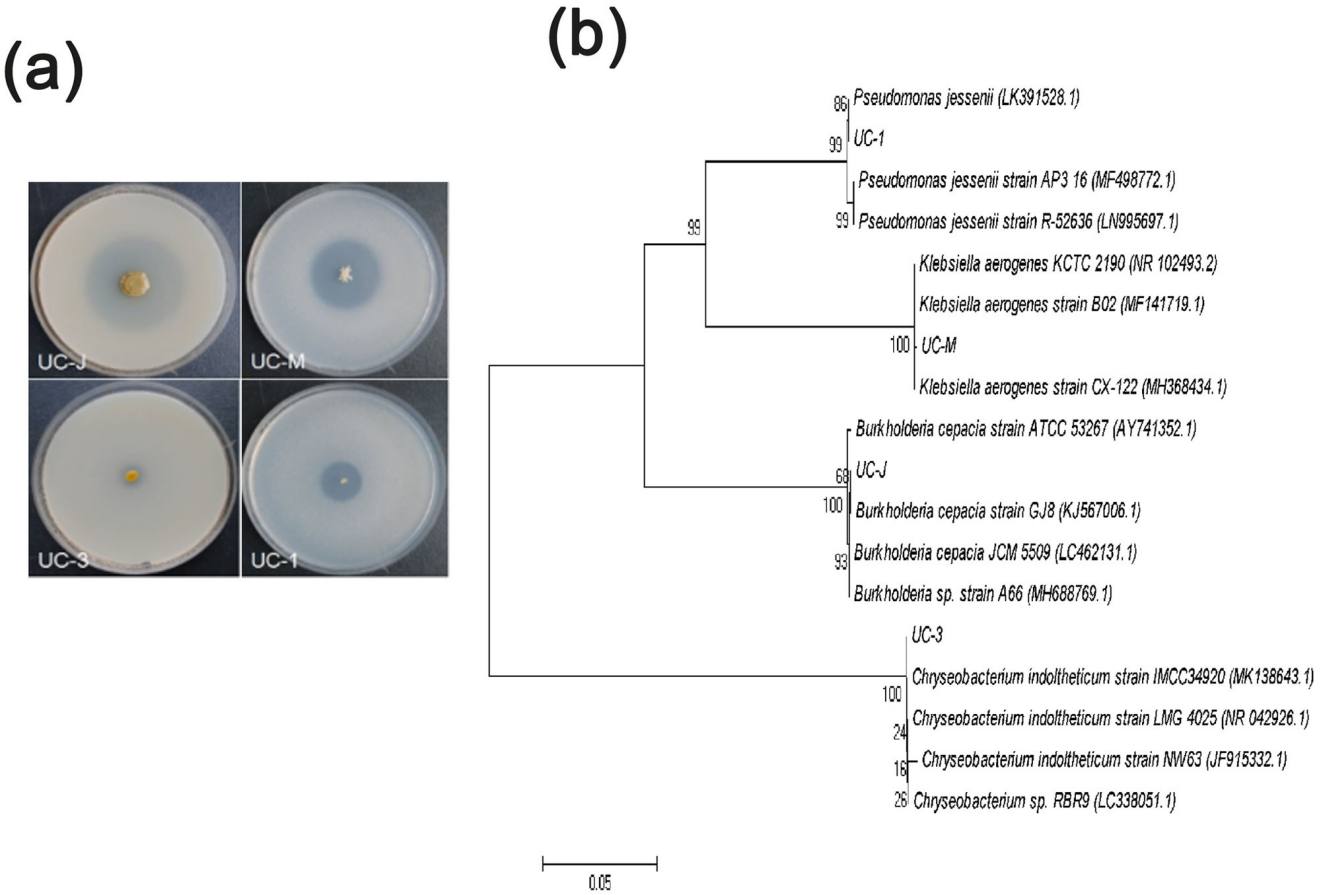
Colony morphology of the four isolates (UC_1, UC_M, UC_J, and UC_3) (a); Phylogenetic tree of the four PSB based on their 16S rRNA gene sequences (b). Abbreviations: UC_J (*Burkholderia sp*), and UC_3 (*Chryseobacterium sp*).

Twenty-three isolates were obtained from Mount Langya in Anhui province (13) and Nanjing Zhongshan Botanical Garden (10) in Nanjing provinces, China. The phosphate solubility index (PSI) varied among isolates, incubation times, and media. Specifically, the PSI ranged from 1.09 to 6.36 on NBRIP plates and from 1.04 to 3.59 on PVK plates supplemented with tricalcium phosphate (TCP). The four most efficient PSB were determined to be UC_1, followed by UC_M, UC_J, and UC_3 based on PSI values of 6.36, 4.62, 3.59, and 1.71, respectively (Fig 1a).

The isolates were positive for phosphate solubilization activity using both NBRIP and PVK broth medium. All four bacterial strains exhibited high phosphate-solubilizing capacity. The two strains with the highest capacities were UC_J and UC_3, with 10.19 and 9.91 mg·L^−1^ of solubilized phosphate, respectively. The amounts of phosphate solubilized by UC_J and UC_3 were 6.7- and 6.5-times higher than those of the CK, respectively.

**Fig 2:**
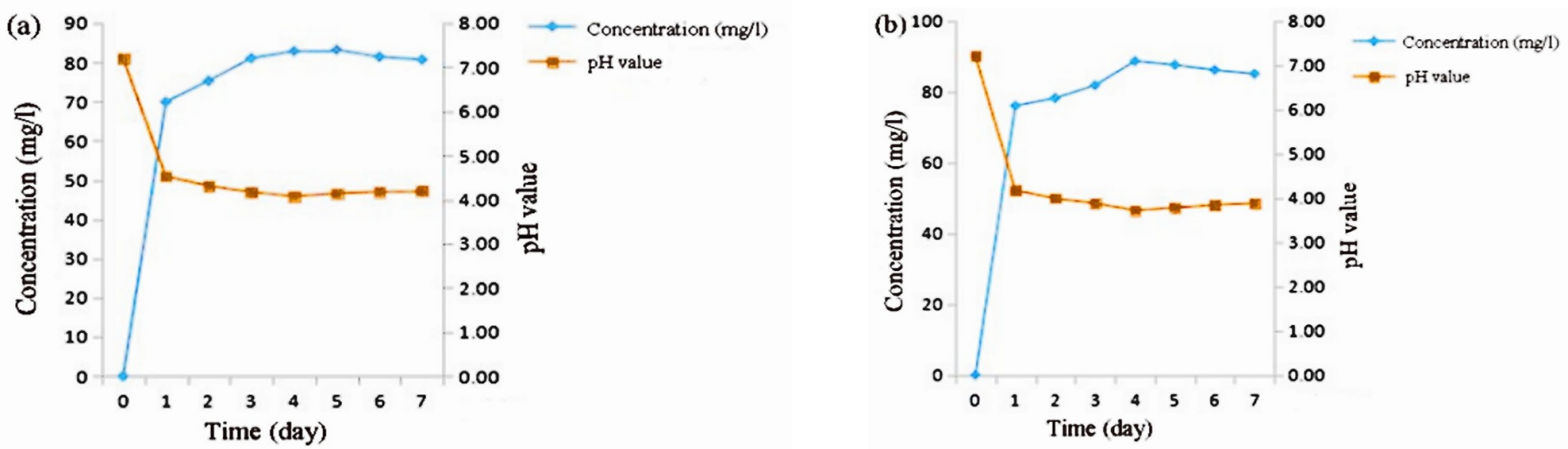
Relationship between phosphorus kinetics and pH changes in response to *Pseudomonas synxantha* (UC-1) (a) and *Enterobacter aerogenes* (UC-M) (b).

Because solubilization of inorganic P is often associated with a reduction in pH by microorganisms, we measured the pH of NBRIP liquid medium inoculated with the inorganic-phosphate-solubilizing bacterial strains exhibiting higher phosphate-solubilization activities. The pH decreased during the first few days of bacterial growth, which was correlated with phosphate solubilization (Fig 2a). The concentration of solubilized phosphate in the filtrate of cultures of the UC-1 isolate increased considerably after 1 d of incubation and then increased gradually from 69.93 to 83.21 mg L^−1^. Additionally, the pH drastically decreased during the course of the experiment, from 7.20 to 4.1. Isolate UC_M had the highest phosphate-solubilizing activity, resulting in the release of large amounts of P into the medium, with levels reaching 88.61 mg L^−1^ after 7 d of incubation. The pH of medium inoculated with UC_M ranged from 7.20 to 3.7 (Fig 2b).

The characteristics of the four isolates are listed in Table 1. Comparison of the 16S rRNA sequences of isolates UC_3 and UC_J to those in the GenBank database revealed that these isolates are closely related to *Chryseobacterium* sp. and *Burkholderia* sp., respectively. The 16S rRNA sequences of the inorganic-phosphorus-solubilizing bacteria isolates were similar to those of *Pseudomonas* sp. (UC_1) and *Klebsiella* sp. (UC_M) (Fig 1b).

**Table 1:** Analysis of 16S rRNA in the four isolates.

Isolate	Species name	% Gene identity
UC_1	*Pseudomonas sp*	100.00%
UC_3	*Chryseobacterium sp*	100.00%
UC_J	*Burkholderia sp*	99.93%
UC_M	*Klebsiella sp*	99.93%

### Production of organic acids by PSB isolates

We detected several low molecular weight organic acids [oxalic acid (OA), malic acid (MA), fumaric acid (FUA), formic acid (FOA), α-ketoglutaric acid (KA), lactic acid (LA), maleic acid (MAA), propanedioic acid (PA), acetic acid (AA), and acrylic acid (ACA)] in the culture supernatants of the four selected PSB (Table 2). As the incubation time increased, the concentrations of individual organic acids secreted by the strains varied. Four organic acids, FOA, KA, LA, and AA, were detected in culture supernatants of UC_1 and UC_M after 2, 4, and 7 days. The concentrations of each organic acid differed among the isolates, with high levels of LA produced by UC_1 (up to 150.09 μg/mL). Notably, high levels of MA were produced by UC_M after 4 d of incubation, but this organic acid was not detected before or after this time point. Other organic acids (PA and AA) were detected in UC_M at relatively high concentrations during incubation (Table 2).

**Table 2:** Production of organic acids in NBRIP broth at 2, 4, and 7 days of incubation.

Organic acids	UC_1 (μg/ml)			UC_M (μg/ml)			CK (μg/ml)		
	2	4	7	2	4	7	2	4	7
Oxalic acid	9.45±0.07	10.15±0.50	8.11±0.54	--	9.33±0.61	--	5.74±1.72	5.94±0.37	5.56±0.39
Malic acid	--	--	2.2±0.02	--	111.79±3.26	--	--	--	--
Fumaric acid	0.39±0.01	0.06±0.00312	0.2±0.001	1.20±0.001	0.61±0.02	0.57±0.002	--	--	--
Formic acid	2115.01±3.25	2399.82±7.64989	2447.21±817.13	74.33±1.21	65.85±1.87	601.75±12.15	18.95±0.61	15.19±0.56	17.74±1.16
α-Ketoglutaric acid	112.15±4.86	147.86±1.7205	150.35±3.97	212.71±5.57	447.17±3.72	693.26±9.09	--	--	--
lactic acid	92.3533±1.56	51.95±3.64308	150.09±3.08	8.87±0.56	1.16±0.01	--	--	--	--
Maleic acid	0.01±0.001	0.06±0.00144	0.02±0.001	0.06±0.002	0.05±0.001	--	--	--	--
Propanedioic acid	3.56±0.002	--	2.03±0.015	8.36±0.58	103.19±4.94	947.76±18.15	--	--	--
Acetic acid	16.14±0.36	4.871±0.0274	13.38±0.44	50.07±1.23	38.74±0.62	133.40±5.16	--	--	--
Acrylic acid	0.00	0.06±0.00121	0.01±0.001	0.26±0.002	--	--	--	--	--
Total amount of organic acid	2349.07±8.84	2614.82±10.01703	2773.6±6.59	355.8±6.96	777.89±7.53	2376.74±18.41	24.69±1.39	21.13±1.99	23.3±0.86

Note: ‘--’ refers to unknown organic acids eluted at the following retention times (min); organic acid content (μg/mL); 2, 4, and 7 represent days.

### Quantification of PSB in the rhizosphere of *U*. *chenmoui* Cheng

To estimate the colonization dynamics of UC-M, UC-3, UC-J, and UC-1 in natural soil, we inoculated *U*. *chenmoui* roots in the natural environment with the Rif-resistant mutants (UC-M, UC-3, UC-J, and UC-1). The bacterial density was highest on the first day of inoculation (0 days). However, the number of PSB colonies decreased to 0.7–1.3x10^7^ g CFU/g dry weight on day 30 (Table 3). These results indicate that the PSB (UC-M, UC-3, UC-J, and UC-1) could stably colonize natural soil.

**Table 3:** Colonization dynamics of phosphate-solubilizing bacteria in *U*. *chenmoui* rhizosphere soil.

Strain	1d (cfu/g)	15d (cfu/g)	30d (cfu/g)
UC-M	6.2±0.61 x10^8^b	2.9±0.39 x10^7^b	1.1±0.01 x10^7^a
UC-3	2.1±0.20 x10^8^c	3.7±0.59 x10^7^a	1.3±0.25 x10^7^a
UC-J	9.3±0.95 x10^8^a	3.3±0.61 x10^7^ab	1.3±0.35 x10^7^a
UC-1	0.3±0.01 x10^8^b	1.8±0.31 x10^7^c	0.7±0.01 x10^7^a

### Effects of the four PSB isolates on plant growth

**Fig 3:**
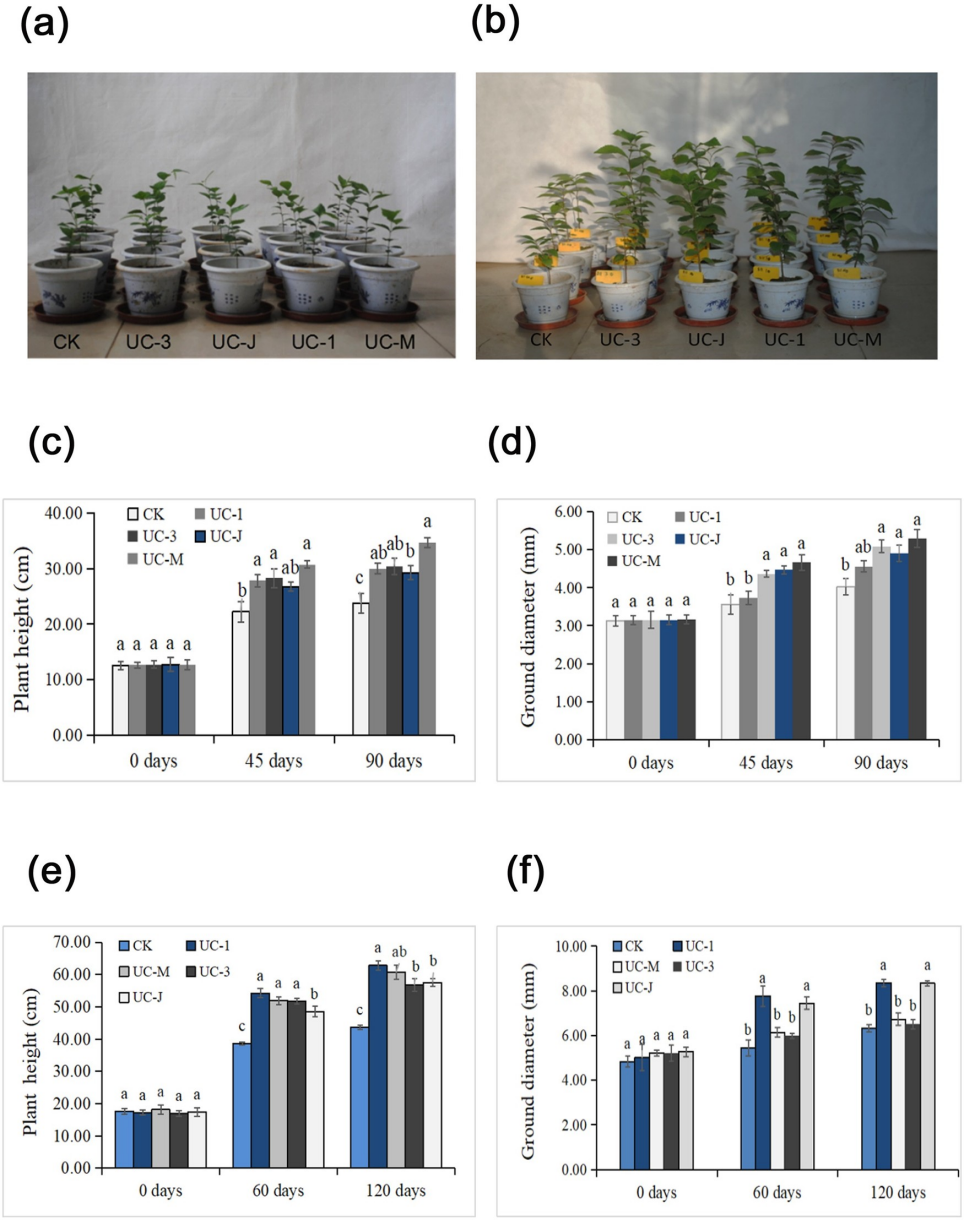
Effects of four phosphate-solubilizing bacteria on the growth of *Ulmus chenmoui Cheng* in the greenhouse. (a) Seedlings inoculated with bacteria on the 1st day; (b) Seedlings inoculated with bacteria on the 90th day. (c) Height of *U*. *chenmoui* seedlings. (d) Ground diameter of *U*. *chenmoui*. Field trial of the effects of the four PSB on seedling height (e) and ground diameter (f) of *U*. *chenmoui* Cheng.

To test whether PSB promote plant growth, we selected the four isolates with the highest P production and determined their effects on *U*. *chenmoui* growth under greenhouse conditions. The seedling height of *U*. *chenmoui* inoculated with any of the four isolates was significantly greater than that of the uninoculated controls. Specifically, UC_1, UC_3, UC_J, and UC_M increased seedling height by 89.04, 137.82, 138.63, and 129.80% (Fig 3c) and ground diameter by 45.07, 61.30, 55.31, and 66.98%, respectively, compared to the control at 90 days after inoculation (Fig 3d). The plant height was significantly greater in the treatment groups than in the control groups at 45 and 90 days after inoculation in the field experiment, at which time the greatest plant heights were 30.78 and 34.70 cm, respectively, as measured in the UC-M group, while the smallest plant heights were 22.72 and 24.57 cm, respectively, as measured in the control group. Isolate UC-M strongly increased the ground diameter (46.97 and 66.98%, respectively) (*p* < 0.05) at 45 and 90 days compared to the control. All PSB inoculants enhanced the seedling height and ground diameter of *U*. *chenmoui* (Fig 3a–3d).

The plant heights of *U*. *chenmoui* recorded in the field at 60 and 120 days after inoculation increased significantly in all four treatment groups relative to the control (Fig 3e and 3f). At 120 days, the maximum plant height of 62.8 cm was recorded for UC_1, while the minimum plant height of 43.63 cm was recorded for the control. Based on these results, UC_1, UC_3, UC_J, and UC_M each significantly improved the growth of *U*. *chenmoui* (*p* < 0.05; Fig 3e and 3f).

### Richness and diversity of the bacterial rhizosphere

After quality filtering and singleton removal, 908,598 16S rRNA sequences remained for community analysis. Across the 15 samples, this corresponds to an average of 60,573 ± 1,568 bacterial sequences per sample. To calculate the diversity index, the OTUs were clustered at a distance of ≤0.03 (approximately 97% sequence similarity). Sequence clustering yielded 2,351 bacterial OTUs (1,420 ± 68.40 per sample) from the five groups (Table 4). These bacterial OTUs were assigned to 1 unique kingdom, 33 phyla, 75 classes, 149 orders, 280 families, 475 genera, and 950 species. In addition, the Good’s coverage values fluctuated between 99.43 and 99.68% (Table 4), indicating that the sequencing depth was sufficient to capture the diversity. Significant differences in the five diversity indices of bacterial community structure were observed between the CK and PSB isolates. The Shannon, Simpson, Chao 1, and ACE diversity indices of bacterial communities in the UC_1, UC_3, UC_J, and CK groups were significantly higher than those in the UC_M group (Table 4).

**Table 4:** Alpha diversity of the root zones of *U*. *chenmoui* at each site.

Samples	Shannon	Simpson	Ace	Chao	Coverage	OTU
UC_3	6.47 ± 0.03 a	0.003 ± 0.0004 b	1630.57± 0.02 a	1644.47 ± 0.05 a	99.68 ±0.20 a	1575.00 ±5.00 a
UC_1	6.25 ± 0.05 c	0.005 ± 0.0003 a	1560.77 ± 0.02 b	1557.48 ± 0.02 c	99.57 ± 0.30 a	1489.67± 20.55 c
UC_J	6.40 ± 0.08 b	0.004 ± 0.0005 b	1556.38 ± 0.06 c	1568.74 ± 0.04 b	99.64 ± 0.40 a	1502.00 ± 2.00 b
UC_M	6.11 ± 0.09 d	0.004 ± 0.0002 b	1557.38 ± 0.01 e	1336.09 ± 0.03 e	99.43 ±0.52 a	1191.00 ± 2.00 d
CK	6.21 ± 0.03 c	0.004 ± 0.0007 b	1558.38 ± 0.07 d	1429.84 ± 0.04 d	99.58 ± 0.25 a	1345.00 ± 1.00 e

Note: UC_1, *Pseudomonas* sp.; UC_M, *Klebsiella* sp.; UC_J, *Burkholderia* sp.; UC_3, *Chryseobacterium* sp.; CK, no PSB treatment. Samples with the same letter within a column are not significantly different (*P* > 0.05).

### Effects of different PSB treatments on bacterial community composition in the rhizosphere

**Fig 4:**
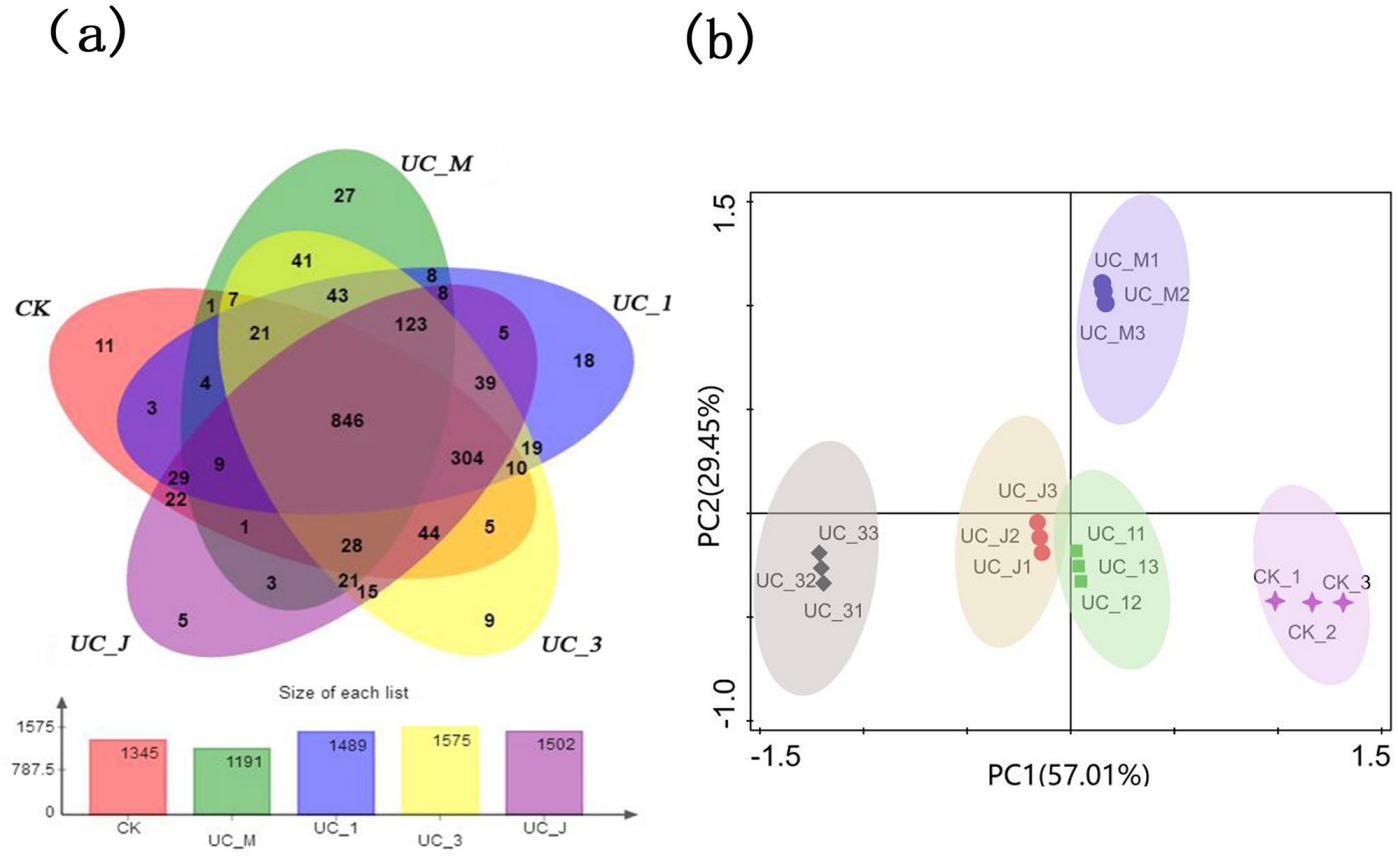
Effects of PSB treatment on plant soil microbiota richness in *U*. *chenmoui*. (a) Venn diagram of four PSB treatments and the CK; (b) Principal components analysis (PCA) analysis of bacteria community in the CK, UC_1, UC_M, UC_J, and UC_3. Symbols on the ordination plot reflect relative dissimilarities in community structures. The variation in microbial community structures explained by each PCA axis is given in parentheses.

As shown in Venn diagrams depicting the number of specific bacterial species (represented by OTUs) associated with the soil samples from the different treatments (Fig 4a), common bacterial species represented more than 90% of the total species recorded. However, a small percentage (5–10%) of bacterial species were specific to the different soil samples. Overall, 1,079 OTUs were common among all soil samples. Moreover, the differences in OTUs demonstrated that each plant rhizosphere had its own bacterial population, and 70 unique OTUs were found within the UC_J, UC_3, UC_1, UC_M, and CK groups (5, 9, 18, 27, and 11, respectively) (Fig 4a).

PCA revealed differences in the bacterial communities: the UC_1 isolate was more similar to UC_J than the others, while the UC_3 isolate was less similar to UC_M than the others (Fig 4b). Furthermore, the bacterial community structure of the CK was different from those of UC_J, UC_3, UC_1, and UC_M. In addition, the first two axes of the PC contributed 57.01% and 29.45% of the variance observed in the clustering, respectively (Fig 4b).

**Fig 5:**
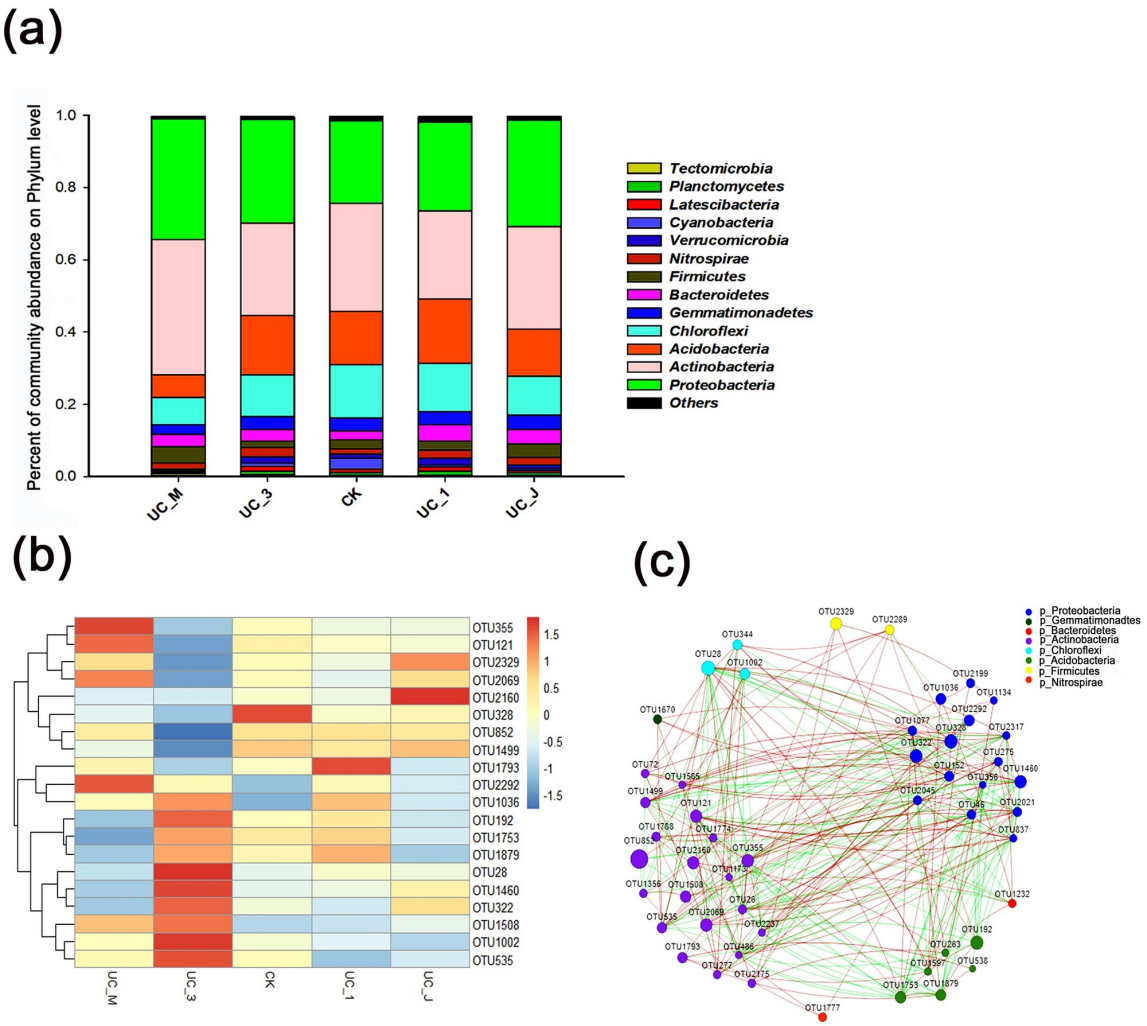
Effects of PSB treatment on plant soil microbiota richness in *U*. *chenmoui*. (a) Relative abundances (%) of the top 13 bacterial phyla present in the five groups. (b) Heatmap depicting the results of hierarchical clustering analysis of the abundance of the main genera in the five different rhizosphere soils. Color intensity of the scale indicates the relative abundance of each OTU read. (c) Co-occurrence networks based on the rhizosphere structural communities of *U*. *chenmoui* plants. Each node corresponds to an OTU, and edges between nodes correspond to positive (red) or negative (green) correlations inferred from OTU abundance profiles using the SparCC method (pseudo *p* < 0.05, correlation values < − 0.5 or > 0.5).

All valid sequences from the soil bacteria libraries were classified from phylum to species based on a BLAST search of the database. Following inoculation of the PSB onto *U*. *chenmoui* plants in the field, we observed differences in bacterial community abundance at different phylogenetic levels (Fig 5a). At the phylum level, we observed 13 phyla of bacteria at relative abundance >1% in the different soil samples collected from the four treatments and the CK: *Proteobacteria (Burkholderiales_bacterium* UC_J UC_M) (24.79–38.5%), *Actinobacteria* (24.23–37.54%), *Acidobacteria* (6.1–17.98%), *Chloroflexi* (7.66–14.74%), *Gemmatimonadetes* (2.64–3.93%), *Bacteroidetes* (*Chryseobacterium* sp. UC_3) (2.32–4.47%), *Firmicutes* (1.69–4.48%), *Nitrospirae* (*s_Pseudomonas_sp*.*_g__Nitrospira; UC_1*) (1.37–2.67%), *Verrucomicrobia* (0.37–1.95%), *Cyanobacteria* (0.36–3.14%), *Latescibacteria* (0.08–1.38%), *Planctomycetes* (0.24–1.15%), and *Tectomicrobia* (0.12–1.01%). Phyla with relative abundances <1% were categorized as “Others” (Fig 5a). The dominant phyla in the soil samples were *Actinobacteria*, *Proteobacteria*, *Acidobacteria*, and *Chloroflexi*. The relative abundance of *Chloroflexi* decreased significantly in response to PSB application in all treated soil samples. However, the change in *Proteobacteria* was opposite that of *Chloroflexi*. The most abundant phylum identified in all treatment groups was *Actinobacteria*, with particularly high abundance observed in the UC_M (37.54±3.56%) group (Fig 5a). The relative abundance of *Gemmatimonadetes* was higher in the CK (3.74±1.05%) and UC_J (3.93±1.03%) groups than in the UC_M (2.64±0.56%) group. Thus, the phylum distribution varied under control and PSB treatments during the early growth of *U*. *chenmoui* (Fig 5a).

In total, 475 genera were detected in all five groups. The relative abundances of these bacteria are shown in Fig 5b and Table 5. *Roseiflexus* (2.54%), *Bacillus* (2.43%), *Nitrospira* (2.27%), *Gaiella* (1.95%), *Flavobacterium* (1.89%), *Streptomyces* (1.15%), and *Solirubrobacter* (1.04%) were the most enriched genera in *U*. *chenmoui* soil. For the PSB (UC_1, UC_3, UC_J, and UC_M) treatment groups, OTU2292 (*Steroidobacter*) levels were primarily enriched, but OTU328 (*Sphingomonas*) levels greatly decreased compared to the CK (Fig 5b, Table 5).

**Table 5:** Bacterial OTU taxa detected at the sample sites.

ID	UC_3	UC_1	UC_J	UC_M	CK	Taxonomy
OTU852	508±15.59	2071±15.59	1721±15.59	2020±15.59	3275±144.58	*g__unclassified_f__Micrococcaceae*
OTU2329	136±13.86	192±13.86	540±13.86	343±13.86	274±2.31	*g__Bacillus*
OTU2069	219±10.39	255±10.39	408±10.39	431±10.39	368±25.51	*g__unclassified_f__Intrasporangiaceae*
OTU355	194±2.31	226±2.31	265±2.31	666±2.31	356±32.33	*g__Blastococcus*
OTU121	118±10.97	237±10.97	238±10.97	640±10.97	384±2.31	*g__unclassified_f__Nocardioidaceae*
OTU1753	326±13.28	375±13.28	240±13.28	13±9.29	375±14.43	*g__RB41*
OTU1879	303±14.43	349±14.43	201±14.43	47±14.43	264±2.31	*g__norank_c__Acidobacteria*
OTU1499	228±14.43	240±14.43	281±14.43	94±14.43	233±3.76	*g__norank_o__Gaiellales*
OTU1002	334±8.08	208±8.08	211±8.08	168±8.08	215±8.66	*g__norank_c__TK10*
OTU535	285±15.01	199±15.01	245±15.01	144±15.01	212±6.93	*g__Gaiella*
OTU1508	322±13.28	243±13.28	286±13.28	273±13.28	188±4.62	*g__Gaiella*
OTU2292	277±6.35	280±6.35	248±6.35	297±6.35	110±5.77	*g__Steroidobacter*
OTU1036	308±13.28	345±13.28	249±20.21	185±13.28	94±3.46	*g__norank_f__Rhodospirillaceae*
OTU1793	233±20.21	341±20.21	211±13.28	136±20.21	172±5.77	*g__norank_c__Actinobacteria*
OTU28	723±25.98	605±25.98	481±25.98	148±25.98	512±6.93	*g__norank_c__KD4-96*
OTU192	485±13.28	589±13.28	286±13.28	57±13.28	587±4.04	*g__RB41*
OTU328	255±10.39	363±10.39	399±10.39	208±10.39	833±19.05	*g__Sphingomonas*
OTU1460	460±20.21	348±20.21	457±20.21	80±20.21	318±10.39	*g__norank_f__Xanthobacteraceae*
OTU322	412±15.01	322±15.01	495±15.01	163±15.01	459±0.58	*g__Bradyrhizobium*
OTU2160	273±14.43	307±14.43	498±14.43	174±14.43	351±0.58	*g__Streptomyces*

Note: This table shows only the relatively dominant OTUs. UC_1, *Pseudomonas* sp.; UC_M, *Klebsiella* sp.; UC_J, *Burkholderia* sp.; UC_3, *Chryseobacterium* sp.; CK, no PSB treatment. Abbreviation: g, genus; f, family; c, class; o, order.

Although the microbiomes changed in ea ch group, a core microbiome was maintained during the growth of *U*. *chenmoui*. Most correlations between the major contributors to the network were positive (Fig 5c, Table 6). *Acidobacteria* had the strongest negatively correlations with *Actinobacteria* and *Proteobacteria*, and *Chloroflexi* had the strongest negative correlations with *Actinobacteria* and *Proteobacteria*. However, *Proteobacteria* were strongly positively correlated with *Bacteroidetes* (Fig 5c, Table 6).

**Table 6:** Pearson correlation among the core bacteria.

Species	Cyanobacteria	Firmicutes	Actinobacteria	Proteobacteria	Bacteroidetes	Nitrospirae	Verrucomicrobia	Acidobacteria	Chloroflexi	Gemmatimonadetes
Cyanobacteria	1	-0.16	0.61	-0.59	-0.55	-0.41	0.08	0.31	0.69	0.50*
Firmicutes	-0.17	1	0.59	0.21	0.06	-0.60	-0.74	-0.65	-0.43	-0.24
Actinobacteria	0.61	0.59	1	-0.31	-0.64	-0.91	-0.69	-0.48	-0.01**	-0.05*
Proteobacteria	-0.59	0.21	-0.31	1	0.32	0.45	-0.18	-0.18	-0.46	0.03*
Bacteroidetes	-0.55	0.06	-0.64	0.32	1	0.65	0.52	0.44	0.16	0.32
Nitrospirae	-0.41	-0.60	-0.91	0.45	0.65	1	0.77	0.67	0.24	0.41
Verrucomicrobia	0.08	-0.74	-0.69	-0.18	0.52	0.77	1	0.95	0.72	0.61
Acidobacteria	0.31	-0.65	-0.48	-0.18	0.44	0.67	0.95	1	0.88	0.81
Chloroflexi	0.69	-0.43	-0.01**	-0.46	0.16	0.24	0.72	0.88	1	0.85
Gemmatimonadetes	0.50	-0.24	-0.05*	0.03*	0.32	0.41	0.61	0.81	0.85	1

Note: Correlation is significant at the 0.05 level. Significance levels: *p < 0.05, **p < 0.01.

## Discussion

In the present study, we examined the roles of the plant-growth-promoting bacteria UC_1 (*Pseudomonas* sp.), UC_M (*Klebsiella* sp.), UC_J (*Burkholderia* sp.), and UC_3 (*Chryseobacterium* sp.) in improving plant growth (Table 1). We obtained these selected isolates from the rhizosphere of *U*. *chenmoui* growing in the Langya Mountain region in Anhui Province and Huashan Mountain in Jiangsu Province, China. Sachdev et al. (2009) [52] showed that *Klebsiella* sp. promoted wheat growth under axenic conditions. Plant growth-promoting bacteria (PGPB) such as *Burkholderia* and *Pseudomonas* have been shown to improve plant growth [53, 54]. *Chryseobacterium* sp. are generally metal tolerant [55, 56], but their roles as phosphate solubilizers have rarely been reported [57]. Interestingly, several *Chryseobacterium* sp. promote plant growth or show biocontrol activity against soil-borne plant pathogens [57, 58]. In the current study, the PSB UC_1, UC_M, UC_J, and UC_3 were inoculated into soil as exogenous phosphate-solubilizing bacteria. Illumina MiSeq analysis showed that these PSB did not dominantly colonize the *U*. *chenmoui* rhizosphere. However, they had strong growth-promoting activities and improved *U*. *chenmoui* growth (Fig 3). Therefore, these PSB (UC_1, UC_M, UC_J, and UC_3) represent potential strains for biofertilizer production.

The aims of this study were to unravel the structures and interactions of rhizosphere microbial communities associated with *U*. *chenmoui* and to determine how introducing PSB (UC_1, UC_3, UC_J, and UC_M) could alter these communities. We demonstrated that inoculation with PSB influenced the rhizosphere microbial communities of the *U*. *chenmoui* trees analyzed, as revealed by analysis of alpha and beta diversity (Table 4). Soil microbial communities drive soil organic matter decomposition and nutrient cycling [59, 60]. We detected a higher Chao 1 index for bacterial community diversity in UC_3, UC_1, and UC_J inoculated rhizosphere soil compared to the CK. Therefore, inoculation of PSB (UC_1, UC_3, UC_J, and UC_M) selected from the *U*. *chenmoui* rhizosphere can improve the fertility of soil containing this plant to a certain extent.

Investigating bacterial communities and diversity in plant rhizospheres is important because microbes have direct beneficial or pathogenic effects on plants. Metagenomic analyses have provided detailed information about microbial diversity, composition, richness, and structure in forests soils [61]. Soil microbial communities are influenced by multiple factors including plant type, climate, and soil properties [62, 63]. However, few studies have focused on the soil community after the in situ use of PSB [64, 65]. In the current study, each plot had uniform terrain and climatic conditions, which allowed us to detect changes in soil microbial communities in response to PSB treatment (Fig 5a). The inoculated PSB did not become the dominant strains in the rhizosphere (Table 5), but they still promoted *U*. *chenmoui* growth (Fig 3). This finding suggests that PSB (UC_1, UC_3, UC_J, and UC_M) treatment could enhance the abundance of beneficial microorganisms and decrease the abundance of harmful microorganisms. In addition, the results show that the effects of colonization of PSB (UC_1, UC_3, UC_J, and UC_M) on soil nutrition, microbial population, and niche were maintained over time, which could affect the invasion of other indigenous bacteria in the future. In addition, PCA (Fig 4b) revealed that the microbial community was greatly influenced by PSB, perhaps because inoculation with PSB (UC_1, UC_3, UC_J, and UC_M) strongly influenced the physical and chemical properties of the soil. Therefore, the artificial synthesis of beneficial microbial communities in the rhizosphere of *U*. *chenmoui* is an important but challenging goal.

The number of microorganisms varied greatly among treatments. The composition of bacteria in the soil could be used as an indicator of the ecological condition of the soil. For example, *Acidobacteria* is one of the most abundant bacterial phyla in terrestrial ecosystems [66] and is found in oligotrophic soils [67]. In the current study, we detected a negative correlation between the relative abundances of *Proteobacteria* and *Acidobacteria* in the *U*. *chenmoui* rhizosphere (Fig 5c, Table 6). *Proteobacteria* have positive effects on plant health [68]. Following inoculation with UC_J and UC_M, the number of *Acidobacteria* groups decreased in the *U*. *chenmoui* biosphere, perhaps due to the biotic stress experienced by *U*. *chenmoui* plants (Fig 5a). These results indicate that PSB UC_J and UC_M improve soil fertility, primarily because these strains dissolve nutrients in the soil that are not available to plants, such as insoluble potassium, iron, and P. The increase in available nutrients in the rhizosphere of *U*. *chenmoui* promoted plant growth. *Actinobacteria* are often found in eutrophic soils [69, 70], and play critical roles in the decomposition of organic matter and humus formation [71]. Inoculation with UC_M induced an increase in the number of *Actinobacteria* (Fig 5a), indicating that the UC_M strain improved soil nutrition.

*Chloroflexi* is abundant in extreme, stressful environments, such as saline water, low-temperature meadow soils, hydrothermally active sediment, oceans [72–79], and geothermal soils [77, 80]. However, in the current study, the number of *Chloroflexi* decreased after inoculation with UC_1, UC_J, UC_M, and UC_3 (Fig 5a), indicating that the conditions were less stressful after PSB inoculation. *Proteobacteria* was the best-represented phylum in all rhizosphere soil samples. Bacteria belonging to this phylum are fast-growing organisms that prefer carbon-rich environments, such as the conditions found in the rhizosphere [70]. In the current study, after the application of all four PSB, the relative abundance of *Proteobacteria* in the rhizosphere of *U*. *chenmoui* significantly improved (Fig 5a). These findings suggest that these organisms promote plant growth by secreting a growth-promoting substance or by releasing a chemical signal to promote their own growth, thereby improving their relative abundance and accelerating plant uptake of nutrients from the soil to promote plant growth. Microbial communities are sensitive, effective, reliable indicators of soil health [81, 82], pointing to the important ecological roles of PSB (UC_1, UC_J, UC_M, and UC_3) in the bacterial community at the Mount Langya Experimental Farm.

Network analysis of taxa co-occurrence patterns offers new insights into complex microbial communities compared to patterns using standard alpha/beta diversity metrics, which are more difficult to unveil [83]. The soil is thought to be one of the most complex environments for microbial life. *Bacteroidetes* can degrade complex organic matter in the biosphere, especially polysaccharides [84] and hydrocarbons in contaminated marine environments [85, 86]. *Proteobacteria* and *Actinobacteria* are abundant in nutrient-rich soils [70], while *Chloroflexi* are abundant in nutrient-poor soils [87]. Hence, the detection of these bacterial phyla and analysis of correlations within a dataset could confirm the nutritional and contamination status of a soil sample. After inoculating the *U*. *chenmoui* rhizosphere with PSB strains UC_1, UC_M, UC_J, and UC_3, we determined that *Chloroflexi* was the most negatively correlated with *Actinobacteria* and *Proteobacteria*. However, *Proteobacteria* was strongly positively correlated with *Actinobacteria* and *Bacteroidetes* (Fig 5c). Analysis of the effects of these four PSB (UC_1, UC_M, UC_J, and UC_3) from the *U*. *chenmoui* Cheng rhizosphere on plant growth and soil bacterial diversity indicated that these PSB could potentially be used to protect plants against abiotic stress, supply limited nutrients to the plants, detoxify soil pollutants, and improve soil conditions.

Given that all bacterial strains used in our study were isolated from the roots of healthy *U*. *chenmoui* plants, the contrasting effects of synthetic communities of bacteria on plant health are surprising. Our data show that the roots of *U*. *chenmoui* in their natural habitats host a rich diversity of bacterial strains, but a single *U*. *chenmoui* root-associated bacterium is not sufficient to ensure plant growth. However, the re-colonization of indigenous bacteria from *U*. *chenmoui* into the most complex multi-microbial consortium resulted in maximum plant growth and survival in our gnotobiotic plant system. Thus, we propose that mutual selective microbial assemblages have, over evolutionary timescales, favored microbe–microbe interactions rather than associations with a single microbial class.

## Conclusions

We isolated and characterized four efficient PSB strains, UC_M (*Klebsiella* sp.), UC_3 (*Chryseobacterium* sp.), UC_1 (*Pseudomonas* sp.), and UC_J (*Burkholderia* sp.), from the rhizosphere of *U*. *chenmoui*. These four strains of PSB might play important roles in the formation of the quality traits of *U*. *chenmoui*. Therefore, in a follow-up study, we plan to re-inoculate the rhizosphere with the dominant strain to study its effects on the growth of *U*. *chenmoui*. We will also explore the potential use of these four strains as microbial fertilizers to improve the rhizosphere microenvironment of *U*. *chenmoui* in other regions.

Overall, pyrosequencing analysis suggested that the addition of individual species of PSB to the roots of *U*. *chenmoui* had various effects on the bacterial community of the rhizosphere, in which certain groups of bacteria were favored to various extents. However, there is still insufficient knowledge to determine the effects of the introduction of bacterial species in the environment and the resulting impact of this practice on soil microbiota. We hypothesize that the introduction of PSB will improve soil fertility via short- or long-term processes. PSB could be used as biotechnological tools to rehabilitate degraded forest soils or to maintain fertility [88]. Identifying indicator species will help determine whether the changes in microbial communities are positive or negative for a functioning forest ecosystem. In particular, the shift we identified in the microbial community of forest soils could, at least partially, be driven by the application of PSB to trees. The extent to which these PSB from the *U*. *chenmoui* rhizosphere also lead to higher rates of soil carbon accumulation merits further studies. When more is known about the resulting ecological alterations in microorganisms in the root zone of the endangered elm species *U*. *chenmoui* following inoculation with PSB, new strategies could be developed to maximize the positive effects of this practice.
